# Selection of reference genes for quantitative real-time PCR analysis in chicken embryo fibroblasts infected with avian leukosis virus subgroup J

**DOI:** 10.1186/1756-0500-6-402

**Published:** 2013-10-07

**Authors:** Falong Yang, Xiaowen Lei, Alexander Rodriguez-Palacios, Cheng Tang, Hua Yue

**Affiliations:** 1 Department of Veterinary Medicine, College of Life Science and Technology, Southwest University for Nationalities, Chengdu 610041, China; 2 Ganzhou Institute of Animal Science, Ganzhou 341401, China; 3 Food Animal Health Research Program, Ohio Agricultural Research and Development Center, The Ohio State University, Wooster, OH 44691, USA

**Keywords:** Avian leukosis virus, Reference gene, Real-time polymerase chain reaction

## Abstract

**Background:**

The selection of stably expressed reference genes is a prerequisite when evaluating gene expression, via real-time PCR, in cells in response to viral infections. The objective of our study was to identify suitable reference genes for mRNA expression analysis in chicken embryonic fibroblasts (CEF) after infection with avian leukosis virus subgroup J (ALV-J).

**Findings:**

The expression levels of 11 potential reference genes in CEF infected with ALV-J were determined by real-time PCR. The expression stability of these genes were analyzed and ranked using the geNorm tool. Analysis indicated that the genes *RPL30* (ribosomal protein L30) and *SDHA* (succinate dehydrogenase complex, subunit A) were the most stably expressed genes in the ALV-J infected CEF.

**Conclusions:**

The *RPL30* and *SDHA* were deemed suitable for use as reference genes for real-time PCR analysis of mRNA gene expression during ALV-J infection, whereas commonly used *ACTB* and *GAPDH* are unsuitable to be reference genes.

## Background

Avian leukosis virus subgroup J (ALV-J) is an avian oncogenic retrovirus that induces myeloid leukosis in poultry, a disease that causes significant economic losses in the broiler breeder industry worldwide. Disease severity of ALV-J is linked to an increase incidence of tumor formation, immunosuppression, with ensuing high mortality rates [1]. To elucidate the molecular pathogenesis of ALV-J infection and tumor development, many studies have focused on analyzing host gene expression profiles following ALV-J infection [2-4]. Assessment of mRNA expression levels via real-time PCR has been a standard approach with high accuracy, sensitivity and reproducibility, and thus it has been widely used to infer host gene expression in response to virus infection [5]. To ensure a reliable result in gene expression analysis, the selection of stably expressed reference gene (or genes) is an important technical prerequisite for each individual experimental setting [6]. This is especially important because even the expression stability of candidate reference housekeeping genes varies across host tissue cells, and virus strains [7,8]. Therefore, the selection of reference genes should ideally be determined for each specific cell type and virus. Chicken embryonic fibroblasts (CEF) are frequently used for propagation of ALV-J. Those fibroblasts have also been used as model cells for the study of host-virus interaction. To identify suitable reference genes for mRNA analysis in poultry, here we present the expression stability of 11 housekeeping genes in CEF after ALV-J infection, and propose stably expressed genes for use as reference genes in ALV-J/CEF settings.

## Findings

**Figure 1:**
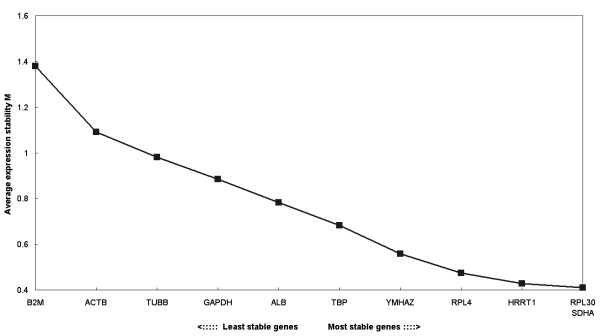
**Expression stability of 11 candidate reference genes.** Expression stability of the reference genes in chicken embryo fibroblasts following avian leukosis virus infection was analyzed by geNorm software. Average expression stability values (M) of the reference genes are plotted from least stable (left) to most stable (right) gene.

The ranking of the 11 candidate reference genes according to their expression stability values (M) is show in Figure 1. From the most stable (lowest M-value) to the least stable (highest M value) genes: *RPL30/ SDHA* < *HPRT1* < *RPL4* < *YHWAZ* < *TBP* < *ALB* < *GAPDH* < *TUBB* < *ACTB* < *B2M*. *RPL30* and *SDHA* have the lowest M value (0.41) and therefore are the most stably expressed genes.

**Figure 2:**
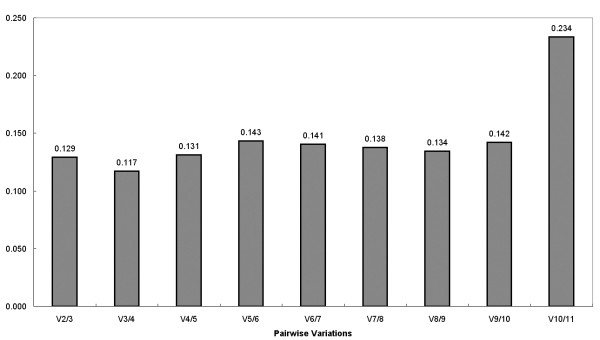
**Determination of the optimal number of reference genes.** The optimal number of reference genes for normalization was determined according to the geNorm software. V_n/n+1_ is the pairwise variation between normalization factors of n and n + 1 genes. Note that although several V_n/n+1_ are below the recommended 0.15 cut off value, it is advisable to choose the set that is composed of the genes that have the highest stability, in this case *RPL30* and *SDHA* for V2/3.

To determine the optimal number of reference genes for accurate normalization, geNorm calculated the pair-wise variation value V_n/n+1_ (Figure 2). It was showed that a combination of the three most stable genes *RPL30, SDHA* and *RPL4* has a lowest pair-wise variation value (V3/4) of 0.117, lower than cut-off value of 0.15. If *RPL4* is excluded, V2/3 = 0.129 is a bit higher than V3/4, but still lower than cut-off value of 0.15. Therefore, two reference genes (*RPL30* and *SDHA*) are sufficient for normalization.

## Discussion

Since the introduction of real-time PCR in virology, RT-PCR has been extremely useful to document host cell responses to virus infection. Although there are several advantages, many factors can affect the performance of the test when quantitating mRNA expression levels. Validation of a given quantitative PCR assay, and that of a normalization process of C_T_ values, is critical to obtain reliable results. The use of “reference genes” as markers of stability has served to normalize variations arisen from differences in nucleic acid integrity, the efficiency of the reverse transcription, and the amount of sample loaded onto a PCR master mix. An ideal reference gene should be one which is stably expressed in cells and unaffected by the experimental treatments.

Viruses, as obligate intracellular parasites, replicate inside cells and use various strategies to induce cell apoptosis, cell transformation, cell death or other dysfunctions by shifting host gene expression on different scales. Therefore, it is reasonable to think that the expression of so-called “housekeeping” genes is probably unstable in virus-infected cells, and that it depends on the virus and host cell types. This highlights the necessity of reference gene selection during real-time PCR analysis for virus-infected cells. Radonić et al. [7] compared the expression of 10 candidate reference genes in cell lines infected with 6 human viruses: cytomegalovirus, human herpesvirus-6, camelpox virus, SARS coronavirus or yellow fever virus, and found that a commonly used gene *ACTB,* is unsuitable as reference gene, whereas TATA-Box binding protein (*TBP*) and peptidyl-prolyl-isomerase A (*PPI*) were stable genes for use as reference genes in expression studies in virus infected cells. However, Yin et al. [9] found that *ACTB* is a stable reference gene in CEF infected with Newcastle disease virus (NDV). Li et al. [10], after comparing 6 housekeeping genes, also showed that *ACTB* is the most stably expressed gene in infectious bursal disease virus (IBDV) infected CEF. Our previous study [11] with CEF infected with H5N1 avian influenza virus demonstrated that *ACTB* and *RPL4* were the most suitable genes for use as endogenous reference genes in avian influenza virus H5N1/CEF settings. In the study carried out by Waston et al. [8], *PPIA* (peptidylprolyl isomerase A), *GAPDH* and *SDHA* were recommended as the best reference genes for host gene expression analysis in cells infection with immunodeficiency virus and herpes viruses.

Although identifying the best reference genes for each type of study may be time and resource-intensive, the studies listed above highlight the need to identify the most stable gene markers for each host virus assay to ensure reliable data. In the present study, *RPL30* and *SDHA* were found to be the most stable reference genes in CEFs infected with ALV-J.

## Conclusions

In conclusion, *RPL30* and *SDHA* could be used as reference genes for the standardization of in CEF gene response to the infection with ALV-J, whereas commonly used *ACTB* and *GAPDH* are unsuitable to be reference genes in ALV-J/CEF settings.

## Methods

All animal experiments were done in accordance with institutional and national ethical guidelines. The protocol was approved by the Ethical Committee for animal experiments of Southwest University for Nationalities.

### Infection of CEF with ALV-J virus

Primary cultures of chicken embryo fibroblasts (CEF) were prepared from 10-day-old specific-pathogen-free (SPF) chicken embryos (Yebio Bioengineering Co. Ltd, Qindao, China) as described previously [12], and maintained in DMEM supplemented with 10% fetal bovine serum (FBS). The cells were seeded (approximately 5 × 10^6^cells/well) in 24-well culture plates. Then the cells were infected with 100 TCID_50_ (50% tissue culture infective dose) ALV-J strain NX0101, obtained from the China Animal Health and Epidemiology Centre in Qindao, China. After 1 h incubation, the cells were washed and further incubated in media with 2% FBS. Host-virus interactions were tested in triplicate wells, which were harvested at 0, 24, 120 and 192 hours after infection for RNA extraction.

### RNA extraction and cDNA synthesis

Total RNA was extracted from each sample using commercial RNAiso Reagent (TaKaRa, Japan) and purified with RNase-free DNase (TakaRa, Japan) following the manufacturer’s instructions. Complementary DNA (cDNA) was synthesized using the PrimeScript® RT reagent (TaKaRa, Japan) with random primers following the product instructions. cDNA products were treated with RNase H to remove remnants of RNA, and stored at-20°C until the time of real-time PCR testing.

### Real-time PCR

A total of 11 housekeeping genes, previously used as references for the evaluation of expression stability in CEF infected with avian influenza virus [11], were also used as candidate reference genes in the present study (Table 1).

**Table 1:** **Primers used in this study [**[11]**]**

**Symbol**	**Gene name**	**Accession no.**	**Primer sequences(5′-3′)**	**Amplicon(bp)**
ALB	Albumin	NM_205261	F: CCTGGACACCAAGGAAAT	197
			R: TGTGGACGCCGATAGAAT	
B2M	Beta-2-microglobulin	NM_001001750	F: CGTCCTCAACTGCTTCGTG	194
			R: TCTCGTGCTCCACCTTGC	
GAPDH	Glyceraldehyde-3-phosphate dehydrogenase	NM_204305	F: AGCACCCGCATCAAAGG	283
			R: CATCATCCCAGCGTCCA	
HRPT1	Hypoxanthine phosphorribosyltransferase 1	NM_204848	F: ACTGGCTGCTTCTTGTG	245
			R: GGTTGGGTTGTGCTGTT	
RPL30	Ribosomal protein L30	NM_001007479	F: GAGTCACCTGGGTCAATAA	160
			R: CCAACAACTGTCCTGCTTT	
RPL4	Ribosomal protein L4	NM_001007967	F: TTATGCCATCTGTTCTGCC	235
			R: GCGATTCCTCATCTTACCCT	
SDHA	Succinate dehydrogenase complex, subunit A	XM_419054	F: CAGGGATGTAGTGTCTCGT	187
			R: GGGAATAGGCTCCTTAGTG	
TBP	TATA box binding protein	NM_205103	F: CGTCAGGGAAATAGGCA	470
			R: GACTGGCAGCAAGGAAG	
TUBB	Beta-tubulin	NM_205315	F: AAAACGAAGTTATCGGGTCTGA	243
			R: ATGCGGCAACCAAATCG	
YWHAZ	Tyrosine 3-monooxygenase/tryptophan 5-monooxygenase activation protein, zeta polypeptide	NM_001031343	F: TCCACCACGACAGACCA	358
			R: CCAGCCTTCCAACTTCC	
ACTB	Beta-actin	NM_205518	F: CTGTGCCCATCTATGAAGGCTA	139
			R: ATTTCTCTCTCGGCTGTGGTG	

Real-time PCR reactions were performed on an ABI 7300 Real-Time PCR Detection System (Applied Biosystems, USA) with SYBR® Premix Ex Taq™ Kit (TaKaRa, Japan). The 20 μl reaction volume consisted of 10 μl SYBR® Premix Ex Taq™, 2 μl cDNA, 0.4 μl ROX reference dye and 0.25 mM of each primer. The following PCR cycling profile was used: one single step at 95°C for 5 min, followed by 45 cycles of 95°C for 30 sec, 56°C for 30 sec, and 72°C for 30 sec, ending with a melting curve analysis from 65°C to 95°C.

### Determination of gene expression stability

To determine the expression stability of 11 candidate genes over CEF cells collected from different time points, the geNorm software [13] was used to calculate gene expression stability measure (M value), which is the mean pair-wise variation for a gene compared with that of all the other tested candidate genes. Genes with higher M values are more variable and therefore have low expression stability. The stepwise exclusion of any given gene with the highest M value allows the ranking of the remaining tested genes based on their increasing expression stability. Candidate genes with the lowest M values have the most expression stability and thus should preferentially be used as reference genes for gene expression and normalization of other genes.

The optimal number of reference genes required for accurate normalization was also determined using geNorm by calculating the pairwise variation value V_n_/_n+1_ between two sequential normalization factors containing an increasing number of reference genes. A large variation means that the added gene has a significant effect and should preferably be included for calculation of a reliable normalization factor. A V_n/n+1_ value of 0.15 has been proposed as a cut-off value, below which the inclusion of an additional reference gene is not required.

## Abbreviations

CEF: Chicken embryonic fibroblasts; ALV-J: Avian leukosis virus subgroup J (ALV-J); FBS: Fetal bovine serum; TCID50: 50% tissue culture infective dose.

## Competing interests

The authors declare that they have no competing interests. This work was supported by the “863” National High-tech Development Research Project (2012AA101304) and Veterinary Medicine Discipline Program of Southwest University for Nationalities (2011XWD-S0906).

## Authors’ contributions

CT and HY created the idea of this study and participated in the design of study. FY designed the primers, participated in data analysis and drafted the manuscript. XL carried out the cell culture, the virus infection and performed the real-time PCR study. AR participated in analysis and interpretation of the data and revised the manuscript. All authors read and approved the final manuscript.
